# Supplementary material for: Human low-density lipoprotein receptor plays an important role in hepatitis B virus infection
Source: PLoS Pathog. 2021 Jul 22;17(7):e1009722. doi: 10.1371/journal.ppat.1009722 (PMC8345860; doi:10.1371/journal.ppat.1009722)
Supplement: S1 Text — (DOCX) [file ppat.1009722.s001.docx]

**Supporting information**

We developed a robust HBV infection cell culture system by overexpressing NTCP in HepG2 cells (HepG2^NTCP^), as previously described [1,2]. This cell culture model of HBV infection was independently validated prior to its use for this study. The input HBV was not detectable by Western blot analysis, as shown by undetectable HBcAg at 24h (1 day) post-infection. However, the levels of HBcAg expression gradually increased over time and reached a plateau at day 5 after HBV infection (S1A Fig). Similarly, HBV infection was demonstrated by immunostaining of HBcAg in the HBV-infected cells as determined by IFA (S1B Fig). The input HBV resulted in a noisy level of HBcAg-immunostaining, which is insignificant compared to the levels of HBcAg expression among uninfected and HBV-infected HepG2^NTCP^ cells at day 5 post-infection (S1B Fig). Taken together, these data demonstrate that HepG2^NTCP^ cell line used in this study support a robust HBV infection and replication. We also used IFA to demonstrate the reduction of HBV infection caused by down-regulation of LDLR expression induced by LDLR-specific siRNAs (S2 Fig), consistent with the results obtained from HBcAg and HBV DNA and cccDNA quantification (Figs 1-9).

**References**

1. Qiao L, Sui J, Luo G. Robust human and murine hepatocyte culture models of hepatitis B virus infection and replication. J Virol. 2018. Epub 2018/09/21. doi: 10.1128/JVI.01255-18. PubMed PMID: 30232184

2. Qiao L, Luo GG. Human apolipoprotein E promotes hepatitis B virus infection and production. PLoS Pathog. 2019;15(8):e1007874. doi: 10.1371/journal.ppat.1007874. PubMed PMID: 31393946; PubMed Central PMCID: PMC6687101.
