# Supplementary figures and images for: Human low-density lipoprotein receptor plays an important role in hepatitis B virus infection
Source: PLoS Pathog. 2021 Jul 22;17(7):e1009722. doi: 10.1371/journal.ppat.1009722 (PMC8345860; doi:10.1371/journal.ppat.1009722)

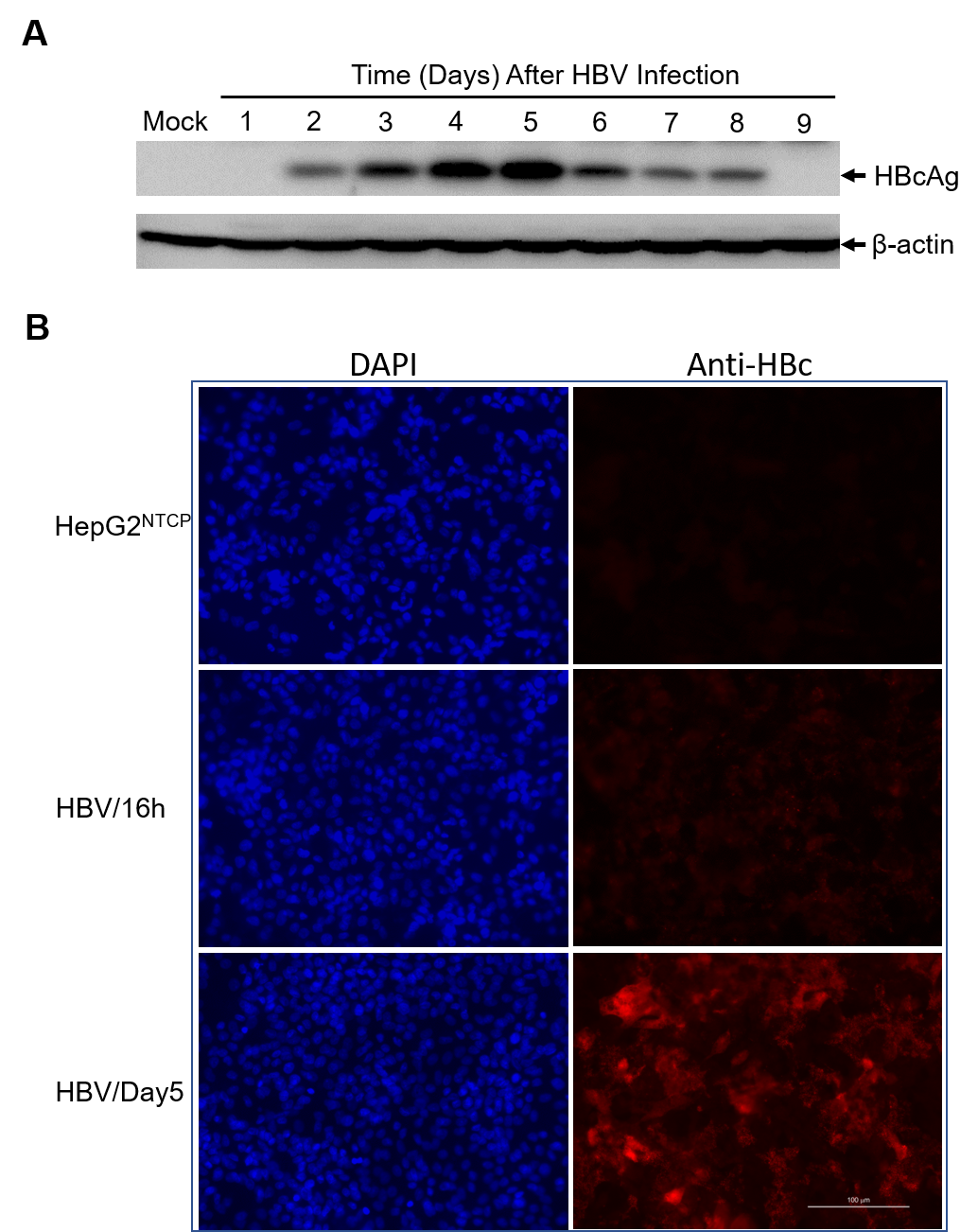

Supplement: S1 Fig — HepG2NTCP cells seeded in 24-well plates were infected with HBV in the presence of 4% PEG. After 12-16h infection at 37°C, the HBV-infected cells were washed with PBS and incubated with DME/F12 medium containing 3% FBS, 1% DMSO, and 5 μg/mL hydrocortisone (HC), as previously described [17]. At different time points (1 to 9 days) post-infection, cells were lysed with a RIPR buffer. A. Detection of HBcAg by Western blot analysis. The lysates of uninfected and HBV-infected HepG2NTCP cells were used for protein separation by electrophoresis in a 10% SDS-PAGE gel. HBcAg and β-actin bands were visualized by Western blotting using an HBc- and β-actin-specific antibodies. B. IFA immunostaining of HBcAg in the HBV-infected cells. IFA is described in material and methods. Uninfected HepG2NTCP cells were used as a negative control. Uninfected and HBV-infected HepG2NTCP cells at 16h (right after HBV incubation as a measurement of input HBV) and day 5 post-infection were subjected to immunostaining of HBcAg by IFA. The nuclei of cells (Blue) and HBcAg (Red) were stained with DAPI and anti-HBc (C1-5)/Alexa Fluor 594 goat anti-mouse IgG, respectively. Fluorescence images were taken using the Nikon S plan fluor 40x objective and either an exposure of 1/3s or 1/2s for DAPI and HBcAg, respectively. Scale bars at the bottom right corner indicate 100 μm. (TIF) [file ppat.1009722.s002.tif]

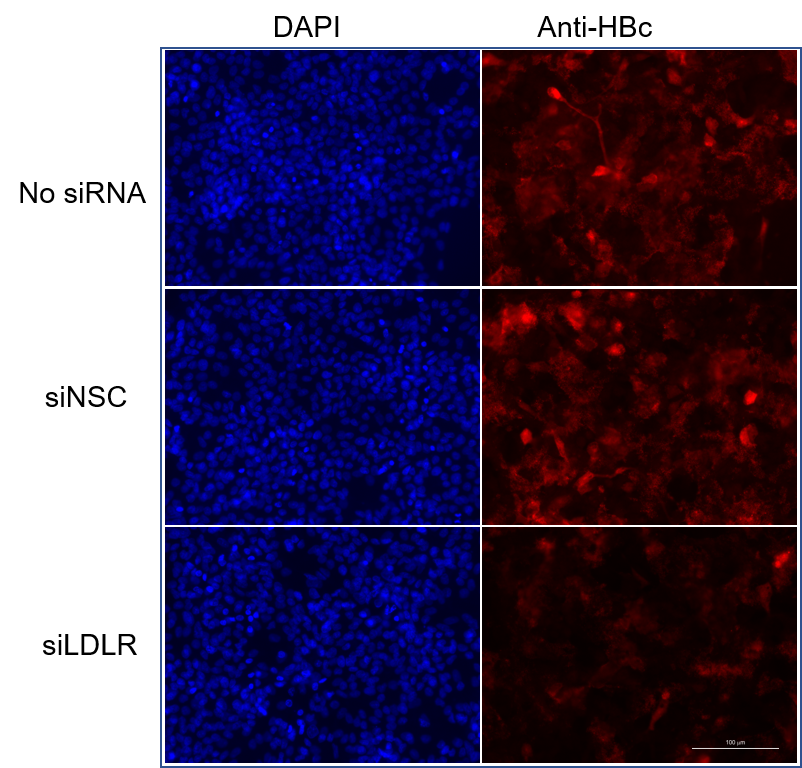

Supplement: S2 Fig — HepG2NTCP cells were transfected with 50 nM of LDLR-specific siRNAs (siLDLR) or a non-specific control siRNA (siNSC) using RNAiMax (Invitrogen). At 48 hours upon siRNA transfection, cells were infected with HBV in the presence of 4% PEG at 37°C for 16 hrs. Uninfected HepG2NTCP (negative control) and HBV-infected cells right after 16-h incubation (input virus control) or at day 5 post-infection were fixed with 4% paraformaldehyde, permeabilized with 0.1% Triton X-100, and stained for HBcAg by IFA. Cell nuclei were stained with DAPI (Blue) and HBcAg was stained with an anti-HBc (C1-5) and the secondary Alexa Fluor 594 goat anti-mouse IgG (red). Fluorescence images were taken using the Nikon S plan fluor 40x objective and either an exposure of 1/3s or 1/2s for DAPI and HBcAg, respectively. Scale bars at the bottom right corner indicate 100 μm. (TIF) [file ppat.1009722.s003.tif]
