# Supplementary material for: Human low-density lipoprotein receptor plays an important role in hepatitis B virus infection
Source: PLoS Pathog. 2021 Jul 22;17(7):e1009722. doi: 10.1371/journal.ppat.1009722 (PMC8345860; doi:10.1371/journal.ppat.1009722)
Supplement: Attachment — Submitted filename: Response to Reviewers-PPATHOGENS-D-21-00109R1.doc [file ppat.1009722.s005.doc]

# Part I – Summary

# SPECIFIC RESPONSE TO REVIEWER #1

We thank reviewer #1 for recognition of our satisfactory response to previous critiques.

# SPECIFIC RESPONSE TO REVIEWER #2

We greatly appreciate the reviewer for acknowledgement of our adequate response to previous review comments.

# SPECIFIC RESPONSE TO REVIEWER #3

We want to thank the review for complementary remarks on the significance of our new findings on the role of LDLR in HBV infection. We understand the reviewer’s concern about the authenticity of HBV infection and have carried out IFA experiments as suggested. These new data are included in two supplementary figures (Fig. S1 and Fig. S2). The input HBV (inoculum) was undetectable by Western blotting as shown by the absence of HBcAg at 24 h post-infection. However, the levels of HBcAg gradually increased over time and reached a plateau at day 5 after HBV infection. Similarly, HBcAg was strongly stained in the HBV-infected cells at day 5 post-infection as determined by IFA. The input HBV resulted in only a noisy signal of HBcAg staining compared to uninfected HepG2^NTCP^ cells, which is negligible compared to strong staining of HBcAg at day 5 after HBV infection (Fig. S1B).

**Part II – Major Issues**

Reviewer #1: None

Reviewer #2: None

Reviewer #3:

To improve the quality of this study, the authors should more precisely describe the HBV particles used for infection. The particles were precipitated with 10% PEG, which is known to also precipitate non-enveloped capsids and nucleocapsids. The virus reparations should be characterized for the presence of LDL, Apo B, Apo A etc. and it should be shown that what is at the origin of HBcAg detection post inoculation does not result from a binding of capsids or nucleocapsids via lipoproteins. Different sources of inoculum would also be required to demonstrate a general phenomenon.

**Response**: We have done HBV purification by Cesium chloride gradient ultracentrifugation as described in our previous PLOS Pathogens paper (Qiao L, Luo GG (2019) Human apolipoprotein E promotes hepatitis B virus infection and production, PLOS Pathog 15(8):e1007874. <https://doi.org/10.1371/journal.ppat.1007874>). HBcAg, HBsAg and apoE were all detected in various fractions. Other apolipoproteins such as apoB and apoAI were also detectable. However, LDLR was not detectable in the HBV preps used in the present study. We do not believe that it is necessary to determine the compositions of HBV preps, which are beyond the scoop of present study and do not have added value to the current study. The origin of HBcAg has been validated by the newly provided data shown in Fig. S1. HBcAg was not detected by Western blotting at 24 h after HBV infection (Fig. S1A) and was negligible as determined by IFA (Fig. S1B). HBcAg detected by Western blotting was from HBV infection and replication rather than the HBV inoculum.

It is equally important that infections be confirmed using immunofluorescence assay for detection of intracellular HBcAg and/or HBsAg in order to measure a percentage of infected cells.

**Response**: We have carried out IFA experiments as suggested. The data are included in Fig. S1B and Fig. S2. Clearly, HBcAg was strongly stained at day 5 after HBV infection (Fig. S1B). The input HBV result in only a noisy signal of HBcAg staining compared to uninfected hepG2^NTCP^ cells but was insignificant compared to HBcAg staining at day 5 post-infection (Fig. S1B). These results are consistent with those obtained from a time-course study of HBV infection. HBcAg was undetectable at day 1 (24 h) after HBV infection but gradually increased to reach a plateau at Day 5 after HBV infection (Fig. S1A). Additionally, siRNA-induced silencing of LDLR expression greatly reduced HBcAg staining (Fig. S2). These newly added data further demonstrate a bona fide HBV infection and replication in the NTCP-expressing HepG2 cells.

To unequivocally proof infection, Northern blotting should be performed as it will detect an HBV product (mRNAs) that is not present in the inoculum.

This reviewer would fully agree with the conclusion of this manuscript if results are confirmed by the suggested experiments.To improve the quality of this study, the authors should more precisely describe the HBV particles used for infection. The particles were precipitated with 10% PEG, which is known to also precipitate non-enveloped capsids and nucleocapsids. The virus preparations should be characterized for the presence of LDL, Apo B, Apo A etc. and it should be shown that what is at the origin of HBcAg detection post inoculation does not result from a binding of capsids or nucleocapsids via lipoproteins. Different sources of inoculum would also be required to demonstrate a general phenomenon.
It is equally important that infections be confirmed using immunofluorescence assay for detection of intracellular HBcAg and/or HBsAg in order to measure a percentage of infected cells.
To unequivocally proof infection, Northern blotting should be performed as it will detect an HBV product (mRNAs) that is not present in the inoculum.
This reviewer would fully agree with the conclusion of this manuscript if results are confirmed by the suggested experiments.

**Response**: I understand the reviewer’s preference of Northern blot for RNA quantification. In general, we prefer Western blot over Northern blot as mRNA products are more convincing. Norther blot requires the use of radioactive materials that we do not have a license at the moment (it takes >6 month to get a license). Additionally, Norther blot does not provide clean bands based on the nature of multiple HBV RNAs and their polyadenylation. Not that many groups like to do Northern blot now. Most groups use qRT-PCR instead of Northern blot for RNA quantification. I hope that quantification of HBcAg and HBeAg by Western blot and Chemiluminescent methods is adequate to support our conclusions.

**Part III – Minor Issues: Editorial and Data Presentation Modifications**

Reviewer #1: No

Reviewer #2: No

Reviewer #3: No
