# Supplementary material for: Human low-density lipoprotein receptor plays an important role in hepatitis B virus infection
Source: PLoS Pathog. 2021 Jul 22;17(7):e1009722. doi: 10.1371/journal.ppat.1009722 (PMC8345860; doi:10.1371/journal.ppat.1009722)
Supplement: Attachment — Submitted filename: Specific response to reviewer comments-PPATHOGENS-S-21-00130.pdf [file ppat.1009722.s004.pdf]

Part I – Summary

SPECIFIC RESPONSE TO REVIEWER #1
We want to thank reviewer #1 for complimentary comments on the data and writing style of our manuscript.

SPECIFIC RESPONSE TO REVIEWER #2
We appreciate the favorable comments of the reviewer on the data that support the important role of LDLR in HBV infection. We agree with the reviewer that the confirmation of virion component(s) interacting with LDLR will have added value to our studies. However, we were unable to pinpoint the exact protein(s) on the viral envelope that interact with LDLR due to the limitation of current HBV production and isolation techniques. Infectious HBV in HBV preparations is just a tiny fraction compared to total numbers of viral and subviral particles. We will wait till a robust HBV propagation cell culture system is developed in the lab to map the molecular interaction between LDLR and HBV envelope proteins. Based on the evidence derived from our study and the findings reported by others, we strongly believe that HBV-associated ApoE mediates HBV cell attachment via interaction with LDLR on the surface of target hepatocytes.

SPECIFIC RESPONSE TO REVIEWER #3
We are surprised by the reviewer's comments that the authenticity of HBV infection is not convincing. Like numerous other independent research groups, we have demonstrated the authenticity of HBV infection in primary human hepatocytes (PHHs) and the NTCP-expressing HepG2 cell lines, as described in two of our previous publications (references 17 and 22). We have used multiple markers (HBcAg, HBeAg, HBV DNA, and cccDNA) of HBV infection to determine the role of LDLR in HBV infection. Input HBV does not contain HBeAg and HBV cccDNA. We have revised and provided detailed information regarding the detection of HBV cccDNA in the material and methods section. Regarding IFA detection of HBcAg, we have tried different antibodies and assay conditions but were unable to exclude the input HBV causing high background signal. IFA is not a good assay for protein quantification unlike Western blot analysis. The LDLR-blocking monoclonal antibody used in HBV infection complicated the use of a highly HBc-specific monoclonal antibody (T2221) for IFA experiments. The rabbit polyclonal antibody specific to HBcAg cross-reacted with many proteins even including human and mouse monoclonal antibodies. Therefore, we are unable to discriminate the newly synthesized HBcAg from the input HBcAg present in HBV viral and subviral particles due to cross-reactivity of rabbit polyclonal antibody. Parental HepG2 is not permissive to HBV infection, as demonstrated by us and many other groups. However, it can be used for HBV attachment assay. We have used the parental HepG2 for HBV cell attachment, as also suggested by reviewer #1. The data from these new experiments are included in the newly added Fig. 9.

Part II – Major Issues

Reviewer #1:

1. Fig. 1 and others, HBc western blot was mainly used in this study for measuring HBV infection. While the HBc western data are largely convincing to support a productive HBV infection, however, the western blot of whole cell lysate could not distinguish the intracellular HBc (internalized and de novo synthesized) from cell bound HBc in the form of inoculated viral particles, if any. Thus, although not absolutely required, an HBc immunostaining of infected cells would provide an additional way to demonstrate HBV infection, which is widely used in HBV field. In addition, Southern blotting of HBV cccDNA and core DNA is a good standard for HBV DNA detection. If possible, the authors may consider this option to make the results more compelling.
Response: We used multiple HBV markers as readouts of HBV infection and replication, including HBcAg, HBeAg, HBV DNA, and HBV cccDNA (Fig. 1, 2, 4, 5, 6, and 7). The HBV-associated input HBcAg could not be detected by Western blot as HBcAg in cell lysates collected prior to day 3 after HBV infection was undetectable (Ref. 17 and 22). In fact, the input HBcAg could be detected by IFA. That is why we used Western blot but not IFA to measure the levels of HBcAg in the HBV-infected cells.

2. Fig. 7, as stated in the manuscript, the purpose of this HBV transfection experiment was to assess the potential effect of LDLR on HBV replication beyond entry events. Hence, it is better to analyze HBV core DNA rather than cccDNA. Moreover, if qPCR is used to detect HBV core DNA or cccDNA, if any, in transfection system, the input plasmid DNA should be eliminated prior to qPCR. In cytoplasmic core DNA extraction, DNase is used to remove non-encapsidated DNA in cell lysate. According to the M&M of this manuscript, only Exo I/III combo was used to treat Hirt DNA before qPCR, which was not able to remove the covalently closed plasmid DNA. Dpn I should be used to fragment plasmid DNA prior to Exo I/III digestion. However, to the best knowledge of this reviewer, a qPCR method for detecting HBV cccDNA in transfected cells has not been reported with validation thus far, and the best evidence supporting cccDNA production in HBV plasmid transfection is the Southern blot data from Jianming Hu's group. If Southern blotting assay is not available to the authors, I would suggest the authors show the core DNA qPCR instead of cccDNA qPCR. Lastly, pCMV-HBV plasmid was used in this experiment, which could not rule out a potential effect of LDLR on HBV core promoter-based transcription. Thus, pHBV1.3 plasmid, which transcribes pgRNA under the authentic HBV core promoter, would serve the authors' purpose better.
Response: The reviewer is correct. Reviewer #2 also raised this concern. We did use DpnI to remove possibly contaminated plasmid DNA. HBV rcDNA was eliminated by treatment of DNA samples with exonucleases I, III, and T5. We could not work with radioactive materials at the moment. It will take a long time to obtain a license at UAB for working with radioactive material. Nevertheless, qPCR has been shown by many investigators as an alternative method for HBV cccDNA detection and quantification although it is not perfect. As suggested by the reviewer, we also determined the levels of HBV DNA upon removal of plasmid DNA by DpnI treatment, which is added as the new Fig. 7C.

3 Fig. 8, the virus-cell attachment assay is normally done under 4°C to block virus internalization, please explain why 37°C was used in this study. In addition, the HepG2 cells (without NTCP) can serve as a clean target cell to specifically assess the binding of HBV to cell upon blocking or depleting LDLR without worrying about NTCP-mediated HBV endocytosis.
Response: This was also raised by reviewer #2. We agree with both reviewers 1 and 2 that virus attachment is normally carried out at 4 °C or on ice. This is true for many other viruses like HCV that we worked in the past. However, HBV attachment and infection are extremely slow, requiring more than 6-hrs for detection of significance levels of HBV attachment and infection. Cells would detach from the plates during PBS washing after more than 3-hrs incubation at 4 °C or on ice. We thank the reviewer for the suggestion to use parental HepG2 cells for HBV attachment assay. The data from these experiments are added as a new Fig. 9 now.

Reviewer #2:

1. There are two alleles of LDLR gene for each cell and the sgRNA targeted sequence for only one allele from each cell clone is represented in Figure 5A. How about another one? It is unlikely that the two alleles are repaired exactly the same way.
Response: These are stable cell lines obtained after a lengthy selection (several weeks). Two alleles of each gene would be separated during cell division. Western blot was used to screen apoE different cell clones for their deficiency of LDLR expression. We only picked up those that did not express LDLR. That is why homogeneous sequence was obtained.

2. Figure 7. in the transient transfection assay, almost all the HBcAg should be translated from pgRNA transcribed from input plasmid DNA and do not reflect the activity of viral DNA replication. In addition, due to the presence of large amount of plasmid DNA, PCR quantification of cccDNA under this experimental condition is difficult and also not necessary. The best way to measure HBV replication in this experiment should be detecting viral DNA replication intermediates by Southern blot hybridization.
Response: We agree with the reviewer that Southern blot (SB) is the best approach for confirmation of HBV DNA and cccDNA. However, we do not have a license to use radioactive materials and cannot perform these experiments in a foreseeable future. Quantitative qPCR has been an alternative as demonstrated by others (Jianming Hu, Stephan Urban, and many others) and is accepted in the field of HBV research. We used the methods described by others to remove plasmid DNA (DpnI digestion followed by T5 nuclease treatment) and rcDNA (exonuclease I and III), prior to qPCR quantification, which are described in the revised manuscript now.

3. For the experiments presented in Figure 8, it will be interesting to see if over-expression of LDLR in HepG2-NTCP cells dose-dependently enhances the attachment of HBV.
Response: Over-expression of LDLR in HepG2-NTCP cells did enhance HBV infection by about 1.8-folds as shown in Fig. 6A (HBcAg) and Fig. 6B (HBV cccDNA).

4. In order to distinguish attachment/binding and internalization of viral particles during infection, the binding assay is usually done at 4 °C to prevent endocytosis or membrane fusion. The current assay condition (37 °C) actually cannot distinguish whether LDLR enhances attachment/binding or internalization. In my point view, this later possibility cannot be ruled out.
<u>Response</u>: The reviewer is correct. For many other viruses like HCV that we worked before, virus attachment assay was carried out at 4°C or on ice. However, HBV infection is extremely slow and requires a lengthy incubation period of time. We have tried 4°C but cells detached from plates after >3 hrs incubation. We could not detect significant levels of HBV under 3hrs incubation. We did acknowledge the possibility of HBV entry when assed at 37°C. To address this shortcoming, we used parental HepG2 cells for HBV cell attachment after silencing of LDLR expression, as suggested by both reviewers #1 and #3. Results from these experiments are shown in the newly added Fig. 9. Indeed, down-regulation of LDLR expression in HepG2 cells also impaired HBV cell attachment.

5. Does purified recombinant ApoE block HBV attachment/binding or infection?
<u>Response</u>: Recombinant apoE does not have good biological activity unless it is assembled as lipoprotein particles. Additionally, recombinant apoE can be internalized quickly as we and others have found previously during our HCV research. Therefore, we do not believe that recombinant apoE will significantly affect HBV infection. However, a circularized apoE peptide (hEP2) may work as it has been shown to block HCV cell attachment. We are going to synthesize this peptide and its mutant control that will be tested by future investigation.

Reviewer #3:

1. an immunofluorescence detection of HBcAg post-inoculation - at 9-12 dpi.
<u>Response</u>: Our previous work had shown that the levels of HBV replication were remarkably decreased after day 6 and were nearly undetectable after day 10 (Fig. 6, JVI 2018, doi: 10.1128/JVI.01255-18, PMID: 30232184). That is why we did not use the conditions (9-12 dpi) as the reviewer suggested. We did try to detect HBcAg by IFA. Unfortunately, the background signals are too high due to cross-reactivity of the rabbit polyclonal antibody specific to HBcAg. The highly specific HBcAg monoclonal antibody T2221 could not be used because C7 is also a mouse monoclonal antibody. We detected higher signal in the C7-treated cells because it bound to LDLR on the cell surface. Nevertheless, we used HBe and HBV cccDNA, both of which are not present in the input HBV, as readouts of HBV infection besides HBcAg. We strongly believe that the detection of HBcAg by IFA will not have much added value. The authenticity of HBV infection in PHHs and hepG2^{NTCP} has been demonstrated not only by us but by many other independent groups.

2. a non-susceptible NTCP-negative cell line (HepG2) should be used as a control.
<u>Response</u>: This is a great idea, which was also suggested by reviewer #1. The parental HepG2 cells were used for siRNA transfection and HBV cell attachment. The data are included as a newly added figure 9 in the revised manuscript.

Part III – Minor Issues: Editorial and Data Presentation Modifications

Reviewer #1:

1. Page 15, the sentence "The genome copy numbers of HBV DNA were determined by a real-time PCR method." needs information of qPCR primers/probe or a reference.
Response: Thanks! We added the information now in the material and methods section. Also, our previous work using these primers and probes is cited.

2. Fig. 1A, would pre-incubation of cells with LDLR antibody achieve a better inhibition of HBV infection?
Response: Pretreatment of cells with C7 antibody had little effect on HBV infection as C7 present during HBV infection reacted with cell surface LDLR quickly and HBV infection requires more than 8 hours for detection of significant levels of HBV replication. We could not detect significant HBV infection when HBV infection (incubation) was less than 4-hrs (Fig. 5A, JVI 2018, doi: 10.1128/JVI.01255-18, PMID: 30232184).

3. Fig.3B, please specify when C7 was added after infection. The loss of inhibition of HBcAg production by C7 after HBV infection actually indicates that there is literally no virus spread in HepG2-NTCP cells, the authors may consider to mention this point.
Response: The reviewer is correct. C7 was added after 12-hrs incubation as indicated in the figure legend now. Current cell culture models used in the lab do not support HBV propagation, as previously shown by us and others. It is sated in the figure legend now (12 hrs). PHHs adapted in humanized mice were shown to support HBV propagation, which will be developed in the lab in the future.

4. Fig. 4, it seems that 2nM siLDLR reduced HBV DNA and HBeAg more obviously than HBcAg. A densitometric analysis of the western bands may help to show the protein levels quantitatively..
Response: We agree with the reviewer. The discrepancy could be due to different sensitivity between Western blot (luminescence) and qPCR (fluorescence). The difference between HBc and HBe reduction at 2 nM is similar (<20% reduction).

5. Fig. 9 needs a non-ApoE negative control.
Response: The apoE-specific monoclonal antibody is highly specific and only reacts with apoE in whole hepatocyte lysates. There is nothing shown up if no apoE is added.

Reviewer #2:

"HBV cccDNA in the cell was extracted by the Hirst method as described previously", it should be Hirt Method.

Response: Thanks! The mistake is corrected now.

Reviewer #3: No
